# Inter-Individual Different Responses to Continuous and Interval Training in Recreational Middle-Aged Women Runners

**DOI:** 10.3389/fphys.2020.579835

**Published:** 2020-10-22

**Authors:** Jèssica B. Bonet, José Magalhães, Ginés Viscor, Teresa Pagès, Josep L. Ventura, Joan R. Torrella, Casimiro Javierre

**Affiliations:** ^1^Secció de Fisiologia, Departament de Biologia Cel⋅lular, Fisiologia i Immunologia, Facultat de Biologia, Universitat de Barcelona, Barcelona, Spain; ^2^LaMetEx–Laboratory of Metabolism and Exercise, Faculdade de Desporto, Centro de Investigação em Atividade Física e Lazer, Universidade do Porto, Porto, Portugal; ^3^Departament de Ciències Fisiològiques, Facultat de Medicina i Ciències de la Salut, Campus de Bellvitge, Universitat de Barcelona, Barcelona, Spain

**Keywords:** middle-aged women, half-marathon runners, high intensity interval training, exercise and sport, moderate intense endurance exercise training, non-responder, responder

## Abstract

A crucial subject in sports is identifying the inter-individual variation in response to training, which would allow creating individualized pre-training schedules, improving runner’s performance. We aimed to analyze heterogeneity in individual responses to two half-marathon training programs differing in running volume and intensity in middle-aged recreational women. 20 women (40 ± 7 years, 61 ± 7 kg, 167 ± 6 cm, and VO_2_max = 48 ± 6 mL⋅kg^–1^⋅min^–1^) underwent either moderate-intensity continuous (MICT) or high-intensity interval (HIIT) 12-week training. They were evaluated *before* and *after* training with maximal incremental tests in the laboratory (VO_2_max) and in the field (time to exhaustion, TTE; short interval series and long run). All the women participated in the same half-marathon and their finishing times were compared with their previous times. Although the improvements in the mean finishing times were not significant, MICT elicited a greater reduction (3 min 50 s, *P* = 0.298), with more women (70%) improving on their previous times, than HIIT (reduction of 2 min 34 s, *P* = 0.197, 50% responders). Laboratory tests showed more differences in the HIIT group (*P* = 0.008), while both groups presented homogeneous significant (*P* < 0.05) increases in TTE. Both in the short interval series and in the long run, HIIT induced better individual improvements, with a greater percentage of responders compared to MICT (100% vs 50% in the short series and 78% vs 38% in the long run). In conclusion, variability in inter-individual responses was observed after both MICT and HIIT, with some participants showing improvements (*responders*) while others did not (*non-responders*) in different performance parameters, reinforcing the idea that individualized training prescription is needed to optimize performance.

## Introduction

One of the general bases of sports training is the principle of individuality, grounded in the specific adaptive responses shown by individuals to a given training program (Matveyev, 1981). This principle of individuality means that the selection or combination of different performance indicators must be carefully chosen to properly assess the training process, since there could be different responses to the same training program (Kenney et al., 2012). A pioneering study by Prud’homme et al. (1984) found considerable individual differences in the adaptive capacity to training in ten pairs of monozygotic twins participating in a short-term endurance training program, with sensitivity to maximal aerobic power being largely genotype-dependent. Later, although similar adaptations in performance and physiological parameters were reported after training schedules involving different exercise volumes and intensities (Gibala et al., 2006; Burgomaster et al., 2008; Scribbans et al., 2014), several studies demonstrated the existence of inter-individual variability in training responses, both after moderate-intensity continuous training (MICT) and high intensity interval training (HIIT; Astorino and Schubert, 2014; Gurd et al., 2016; Whipple et al., 2018).

Participation in half-marathon races has increased across years, especially for the middle-aged female runners (Knechtle et al., 2014, 2016; Knechtle and Nikolaidis, 2018). For example, data from races held in Switzerland between 1999 and 2014 indicate that more endurance athletes compete in half-marathon than in marathons, and that the finisher’s men-to-women ratio decreased significantly throughout the years, meaning an increase in women’s participation (Knechtle et al., 2016). It is also interesting to note that, between 2014 and 2016 in the world’s largest half-marathon (GöteborgsVarvet), approximately 44% of the women’s runners were in the middle-aged group (35 to 50 years; Knechtle and Nikolaidis, 2018). Although amateur running is a leisure activity that has become increasingly popular in recent decades (van Dyck et al., 2017), there are still a few studies focusing on middle-aged non-elite women recreational runners (Machado et al., 2011). Only recently, some studies have been centered on middle-aged female marathon recreational runners, highlighting the effect of sex and age on pacing (Nikolaidis et al., 2018), and reporting new data on anaerobic power and neuromuscular fitness for this population group (Nikolaidis and Knechtle, 2018). These findings are of practical relevance for practitioners and coaches, considering their implications in racing times. Thus, to determine the variability (inter-individual differences) in the responses to different training programs among middle-aged women who normally participate in recreational running would be useful for optimizing strength and pacing times and hence improving health-related physical fitness and race finishing times.

During the last decade, several schedules for preparing half-marathon have been published including different strategies regarding the volume and the intensity of the training. There are programs prioritizing MICT with maximal weekly running volumes of 40 km and long run sessions of 25 km (Galloway and Galloway, 2012), contrasting to those where HIIT and fast pace short distance runs predominate (Lanier et al., 2012). Other schedules alternate high-volume programs with speed and hill training (Hamilton and Sorace, 2018) or with resistance training (Hagerman, 2005). A recently published study from our laboratory (Bonet et al., 2020) was aimed to assess if a mixed program, with HIIT sessions of 40 s to 90 s followed by short recovery periods and interspersed with low-volume endurance sessions, was better for training a half-marathon than a traditional MICT program, based on high-volume endurance sessions of moderate-intensity training. We reported a detailed description of the training schedules, data on the performance and physiological changes elicited by each training program.

Here, we present a preliminary brief research report focused on the analysis of the heterogeneity in individual responses to the training programs. We analyze some parameters that could affect performance and could induce different responses depending on the training program. Our hypothesis was that the HIIT program would result in a more homogeneous response than the MICT program, since HIIT would activate more skeletal muscle metabolic ways and promote greater changes in cardiovascular structure and function. To study the inter-individual variation in response to training is of interest because it has been reported that individuals who fail to respond to an endurance exercise protocol may respond to other training protocols, such as resistance or interval training (Hautala et al., 2006; Bonafiglia et al., 2016). Identifying responders and non-responders to a given training protocol would allow creating individualized pre-training schedules and improve runner’s performance. This would be especially interesting in the population group studied here: middle-aged women, who run at a recreational level, normally having difficulties in combining sports practice with daily professional and family life (Macias et al., 2014). As suggested in a recent review (Pickering and Kiely, 2019), more research is needed to identify responders and non-responders to exercise interventions so that alternative training schedules can be developed for non-responders to increase their fitness in an effective manner.

## Materials and Methods

### Subjects

A total of 20 women aged 40 ± 7 years, 61 ± 7 kg, and 167 ± 6 cm with a body mass index of 23 ± 3 (mean ± SD) participated in this study. They were recruited from different running clubs in the city of Barcelona (Spain) after completing a questionnaire and an interview to assess the following inclusion criteria: to be pre-menopausal, non-smokers, to have no injuries and not to take any medication (including oral contraceptives). Moreover, they age range must be between 35 and 45 years and be regular runners, training a minimum of 5 h and 3 days per week and with previous experience in running half-marathons recreationally. The women were randomly divided into two training groups (*n* = 10 in each): (1) MICT group, which participated in a high-volume and low-intensity training program; and (2) HIIT group, which completed a low-volume and high-intensity interval running program with bodyweight resistance exercises. No significant statistical differences were observed between randomly selected groups in performance parameters prior to training (Table 1). There were no significant differences in the anthropometrical parameters between the groups both at the beginning and at the end of the training protocols. After being informed about the experimental procedures, as well as their risks and benefits, the participants signed an informed consent form and were free to withdraw from the experimental protocol at any time. The study was developed in accordance with the Declaration of Helsinki concerning the ethical principles of human experimentation and was approved by the Institutional Ethics Committee of the University of Barcelona (Institutional Review Board number, IRB00003099).

**TABLE 1:** Finishing times for the half-marathon and results from the maximal incremental tests performed in the laboratory (VO_2_max) and on the athletic track (field time to exhaustion, TTE) *before* and *after* the training programs.

	**Whole group**			**MICT**			**HIIT**			
**Finishing time (h:min:s)**	**Mean**	**Range**	**CV (%)**	**Mean**	**Range**	**CV (%)**	**Mean**	**Range**	**CV (%)**	***P*-value MICT *vs* HIIT**
Before	1:59:36	0:45:33	9.4	2:02:27	0:32:51	7.1	1:56:45	0:36:23	11.3	0.268
After	1:56:26	0:36:54	8.4	1:58:37	0:23:40	7.0	1:54:16	0:36:54	9.6	
*P*-value	0.085			0.298			0.197			

**VO_2_max (mL⋅kg^–1^⋅min^–1^)**	**Mean**	**Range**	**CV (%)**	**Mean**	**Range**	**CV (%)**	**Mean**	**Range**	**CV (%)**	***P*-value MICT *vs* HIIT**

Before	47.9	18.3	11.6	46.1	14.6	10.5	49.7	16.2	11.9	0.153
After	46.0	18.7	11.3	45.8	13.7	9.8	46.1	18.7	13.2	
*P*-value	0.05			0.833			0.008			

**TTE (h:min:s)**	**Mean**	**Range**	**CV (%)**	**Mean**	**Range**	**CV (%)**	**Mean**	**Range**	**CV (%)**	***P*-value MICT *vs* HIIT**

Before	0:15:59	0:09:17	14.2	0:16:19	0:06:26	12.7	0:15:38	0:09:11	15.8	0.508
After	0:16:36	0:09:52	13.3	0:17:06	0:07:16	13.4	0:16:06	0:07:27	13.1	
*P*-value	0.002			0.035			0.029			

*Results are shown for the whole group consisting of all the women participating in the study, as well as separately for the moderate-intensity continuous training (MICT) and high-intensity interval training (HIIT) groups. Data are given as the mean, range and coefficient of variation (CV). *P*-values in the rows show the statistical significance of the differences between *before* and *after* conditions, as determined using paired Student’s *t*-test. *P*-values in the last column show the absence of statistical significance between randomly assigned participants to MICT and HIIT programs prior to the training.*

### Training Programs

Both training programs lasted for 12 weeks, involving 3 non-consecutive sessions per week, from September to December after a 1-month rest in August. For a detailed explanation of the training programs, see Bonet et al. (2020). After the training programs, all the women participated in the same half-marathon race held in Vilanova i la Geltrú (Spain), located at sea level on the Mediterranean coast (41°13’27” N, 1°43’33” E). The race had a mostly flat profile with slight ups and downs between kilometer 9th and 10th and from the 20th to the finish line. Weather conditions during the race were sunny and windless with a temperature of 15°C.

#### Moderate-Intensity Continuous Training

The MICT group followed a training program based on the one described in Galloway and Galloway (2012). This consisted of 2 days of continuous running (40 min and 60 min) and 1 day alternating between long-distance running (from 12 km to 25 km) and 800-meter intervals every week, that were run in approximately 4 min. The total distance trained was approximately 383 km and the overall time invested, calculated as an average among all the participants, was 40 h and 30 min.

#### High-Intensity Interval Training

The HIIT group participated in a weekly training program that was designed to reduce training volume and increase training intensity. The program consisted of a first session of long-distance running (from 8 km to 16 km), a second day dedicated to interval running (200-m and 400-m series), and a third day that alternated between an uphill run and a fast run with bodyweight resistance exercises. The speed of the long-distance running on the first day was calculated for each subject based on their percentage of VO_2_max. The 200-m or 400-m series on the second day were grouped into 1, 2, or 3 blocks, with recoveries between series of the same block ranging from 30 s to 1 min and the recoveries between blocks lasting 3 min. The uphill run on the third day of the week consisted of climbing 100-m 10% to 12% slopes at an intensity of 85% VO_2_max, which was followed by running downhill at a slow pace and then a 10-min fast run at 80% VO_2_max. This was combined with a circuit of 12 stations of weight resistance exercises performed at maximum intensity, based on the training described in Klika and Jordan (2013). When the circuit was completed, 4 50-m sprints and 3 30-s sets of Bosco’s countermovement jump tests (Bosco et al., 1983) were performed in order to improve anaerobic power and increase the efficiency of using elastic energy. The total distance trained by the HIIT group was approximately 301 km and the overall time invested, calculated as an average among all the participants, was 33 h and 26 min. Thus, the women in the HIIT program covered 21% less distance and invested 17% less time than those in the MICT group.

### Maximal Incremental Tests

#### Laboratory

All subjects performed two University of Montreal Track Tests (Léger and Boucher, 1980) in the laboratory (L-UMTT) on a treadmill (Quasar h/p Cosmos^®^ Sports & Medical GmbH, Nussdorf-Traunstein, Germany) to determine the maximal oxygen uptake (VO_2_max). The first test was performed during the first week, prior to the beginning of the training programs; and the second during the last week, prior to the half-marathon race. Briefly, the L-UMTT consisted of: (1) an initial 4-min warm-up period at a speed of 6 km⋅h^–1^ and a slope of 0.6°; (2) an incremental phase with increases of 1 km⋅h^–1^ every 2 min until exhaustion; and (3) a 6-min recovery period that involved sitting down to rest. This test was preceded by a resting electrocardiogram (ECG; CardioScan v. 4.0, DM Software, Stateline, NV, United States) and a 5-min standing rest to determine the baseline VO_2_ with an automated gas analysis system (TR-plus Metasys, Brainware SA, La Valette, France) equipped with a two-way mask (Hans Rudolph, Kansas, United States).

#### Field

To provide a follow-up to the training process and adjust the training loads, two field UMTTs (F-UTTM) were conducted on an official athletics track. Field time to exhaustion (TTE) was recorded at the end of each test. The first test was performed prior to the beginning of the training programs, separated by at least 72 h from the first L-UMTT to avoid the effects of residual fatigue on performance. The second F-UMTT was conducted at the end of the 8th week of training. Cones were set at 50-m intervals along the track and the speed of each stage was controlled by an examiner equipped with a whistle and a chronometer, who emitted sounds when the subjects had to pass a cone in order to maintain a constant speed for each stage of the test. The test finished when the participant was either behind a cone on three consecutive occasions or when she could no longer keep the pace required to pass the cones and decided to stop the exercise.

Two field tests, at the beginning and at the end of the training periods, were performed to assess power performance in short-run series (200 m and 400 m for HIIT and 800 m for MICT) and the long run pace (8 km for both training programs). The first short-run series test (*before*) was performed during the first week (200-m and 800-m series) and second week (400-m series) of training, whereas the second test (*after*) was conducted during the tenth (200-m and 800-m) and eleventh (400-m series) week. The long run tests were performed for both training programs during the first (*before*) and tenth (*after*) week of training.

### Statistical Analysis

We powered the sample size on the variable finishing time to fit the power parameters of α = 0.05 and β = 0.20, estimating both the size of the change to be detected and the size of the standard deviation change as 0.05. A minimum sample size of *n* = 10 was required for paired *t*-tests (comparing *before* vs *after* parameters for each training program). Since an initial sample of twenty-two participants was selected during the recruitment procedure, the study began with eleven participants in each group. However, one woman from each group failed to follow the complete training schedule. Thus, the final sample contained 10 women per group. After checking normality (Kolmogorov-Smirnov test) and homoscedasticity (Levene test), to evaluate changes in the performance indicators, we used paired Student’s *t*-tests (comparisons: *before* vs *after*). One-way ANOVA was run to evaluate intragroup differences in the finishing times between the different types of responders. *P*-values are given throughout the text, tables and figures, considering significant statistical differences at *P* < 0.05. To evaluate variability in the responses to the training programs, we used the coefficient of variation (CV), i.e., the ratio between the standard deviation and the mean (expressed as %). This parameter is normally used to assess variability in a group’s response to a stimulus such as a training program. All data were statistically analyzed using SigmaPlot 11 (Systat Software, Inc., San Jose, CA, United States, 2008–2009).

## Results

### Half-Marathon Finishing Times

After completing the training period, the mean finishing time obtained in the half-marathon by the whole group (i.e., irrespective of the training schedule) showed a non-significant (*P* = 0.085) improvement (Table 1). The previous finishing times were obtained in the half-marathon held 10 months before in Granollers (Spain, 41°36’30” N, 2°17’20” E), with a similar profile, 145 m of elevation and similar weather conditions. This improvement consisted of a 2.4% reduction compared to the mean of previous finishing times, indicating a decrease of 3 min and 10 s compared to the mean of previous finishing times. However, when the data were analyzed by considering the training programs separately, the MICT and HIIT groups behaved differently.

**FIGURE 1:**
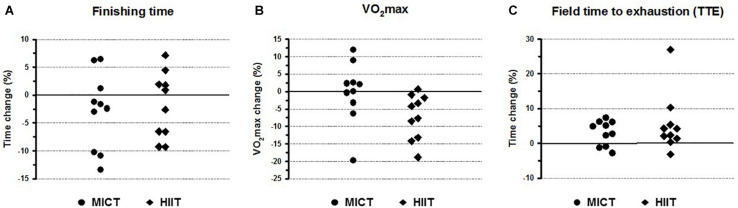
Individual percentage changes *before* and *after* the training program in women participating in moderate-intensity continuous training (MICT) and high-intensity interval training (HIIT). **(A)** percentage changes in time comparing previous (*before*) finishing times and the finishing times registered in the half-marathon race *after* the training programs. **(B)** percentage changes in VO_2_max obtained in a laboratory maximal incremental test *before* and *after* the training programs. **(C)** percentage changes in time to exhaustion (TTE) obtained in a field maximal incremental test *before* and *after* the training programs.

The MICT group showed a non-significant (*P* = 0.298) 2.9% reduction compared to its mean of previous finishing times (Table 1), indicating that it took them 3 min 50 s less to complete the half-marathon compared to previous times. The times before and after the training program showed a similar intragroup variability (CV∼7%), with more than a 9-min difference in the range of the times obtained in the half-marathon after the training protocol. Among the women that followed this training program, three did not improve on their previous finishing times, while another three achieved a time reduction greater than 10% (Figure 1A). Thus, regarding their response to the MICT program, the participants were classified into three categories showing significant differences (*F* = 159.9, *P* < 0.001) in their mean finishing times: (i) three subjects were *high responders*, with a mean time reduction of 15 min; (ii) four subjects could be considered *normal responders*, achieving a mean decrease in their finishing time of 2 min 26 s; and (iii) three subjects were *non-responders*, increasing on their mean previous finishing time by 5 min 31 s.

The HIIT group showed a non-significant (*P* = 0.197) 1.8% decrease compared to its mean of previous finishing times (Table 1), which meant that it took them 2 min 34 s less to complete the half-marathon compared to their previous finishing times. The times obtained *after* the training program showed a slight reduction in intragroup variability (CV = 9.6%) compared to the times obtained *before* the training (CV = 11.3%), but with a similar range difference of ∼36 min in both cases. The women in the HIIT group were classified into two categories (Figure 1A) showing significant differences (*F* = 38.2, *P* < 0.001) in their mean finishing times: (i) five were *responders*, with a mean time reduction of 7 min 54 s compared to the mean of their previous finishing times; and (ii) five were *non-responders*, increasing on their mean previous finishing time by 3 min 37 s.

### Maximal Incremental Tests

When considering the whole group, the VO_2_max recordings (mL⋅kg^–1^⋅min^–1^) obtained in the laboratory maximal incremental tests (L-UMTT) after the training protocols showed a significant decrease of 3.7% (*P* = 0.05) compared to the values obtained before the training started (Table 1). However, when the training groups were analyzed separately, VO_2_max did not show significant differences (*P* = 0.833) *before* and *after* the training program in the MICT group, whilst a significant decrease of 7.2% (*P* = 0.008) was observed in the HIIT group. Moreover, there was greater variability in VO_2_max after the training program in the HIIT group, which presented a larger CV (13.2%) and range (18.7 mL O_2_⋅kg^–1^⋅min^–1^) than the MICT group (CV = 9.8%; range = 13.7 mL O_2_⋅kg^–1^⋅min^–1^). Figure 1B shows that half the women in the MICT group increased their VO_2_max, while the other half of the group decreased their VO_2_max. By contrast, almost everyone (9 out of 10) in the HIIT group decreased their VO_2_max.

Regarding the maximal incremental test performed in the field (F-UTTM), the whole group significantly increased (*P* = 0.002) the TTE in the 8th week of training compared to that recorded in the F-UMTT prior to the start of the training programs (Table 1). When the training programs were considered separately, the TTE significantly increased in both the MICT (4.7% increase) and HIIT (3.0% increase) groups (Table 1). Variability in this performance indicator after the training programs was similar in both groups, with almost the same CV (∼13%) and range (∼7 min). However, there were some differences between the groups in the responses, since three participants in the MICT group failed to improve their times, whilst only one from the HIIT group worsened on her previous times (Figure 1C).

### Field Training Tests

**FIGURE 2:**
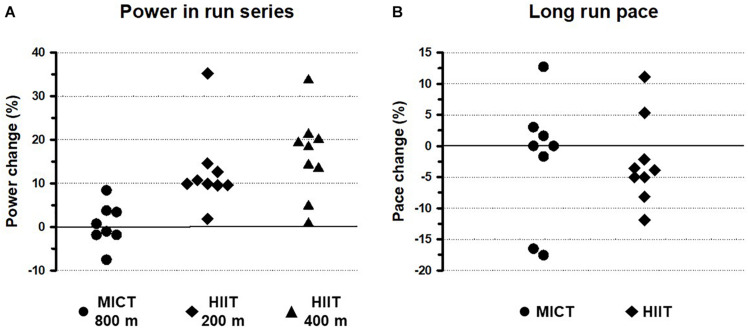
Individual percentage changes *before* and *after* the training program in women participating in moderate-intensity continuous training (MICT) and high-intensity interval training (HIIT). **(A)** Percentage changes in power obtained in interval run series in the field *before* and *after* the training programs. **(B)** Percentage changes in the long run pace (8 km) in the field *before* and *after* the training programs.

Figure 2 shows the percentage variations in individual field tests performed to assess (i) the interval training power output in the 800-m series (MICT program) and in the 200-m and 400-m series (HIIT program; Figure 2A), and (ii) the long run pace over 8 km (Figure 2B).

The women in the MICT group showed varying improvements in their power output in the 800-m interval series. Approximately half of them increased their power output after the training program, while the other half did not increase or even decreased it. This was in contrast to the clear improvement in all the women in the HIIT group for both the 200-m and 400-m series, with mean increases of 9% and 20%, respectively.

The mean long run pace over 8 km showed a 2% to 3% non-significant decrease, i.e., less time was invested per km. Moreover, the analysis of individual results for this field test (Figure 2B) indicated that, overall, the women in the HIIT group showed a better response to training than those in the MICT group (78% vs 38% improved their pace).

## Discussion

Our study examined responses to two training programs involving different intensities and running volumes, both performed 3 days per week over 12 weeks. A global improvement in field performance was observed at the end of the training period for the two training groups. However, there were different inter-individual responses after the training period, with some participants showing improvements in performance indicators (*responders*), while others failed to exhibit positive changes (*non-responders*).

Regarding the finishing times in the half-marathon, the MICT group exhibited a wider range in their responses, with more responders (70%) in this group than in the HIIT group (50%; Table 1). The fact that there were responders and non-responders in both groups (Figure 1A) indicates that the adaptive response to the training programs varied individually, even if the participants followed a standardized and supervised endurance training program with prescribed intensities based on their percentage of VO_2_max. This finding in middle-aged women is in accordance with those of studies on young women (McPhee et al., 2010) and men (Vollaard et al., 2009; Scharhag-Rosenberger et al., 2010). These studies concluded that it is advisable to standardize exercise intensity using other measures related to performance power output, such as blood lactate, rather than the percentage of VO_2_max, due to the inhomogeneous blood lactate responses obtained after prolonged endurance exercise at given percentages of VO_2_max. This is especially relevant in advanced age, as has been reviewed by Lepers and Stapley (2016), who reported that VO_2_max seems to be the parameter most altered by age, contrasting to exercise economy and the lactate threshold, which decline to a lesser extent with advancing age. Taking into consideration this variability in the responses, an individualized exercise prescription might be needed instead of standardized methods. Some proposals have been made, such as those based on kilocalories expenditure per week in relation to body mass (Weatherwax et al., 2016).

Regarding VO_2_max, it must be noted that we found a decrease at the end of the training programs, which was significant in the HIIT group (Table 1). Moreover, all the participants in the HIIT group were *non-responders* for this performance indicator (Figure 1B), suggesting a worsening in performance. However, this could not be the case, especially at high loads, if other performance variables, such as running economy, were improved. Silva et al. (2017) reported that a HIIT program mediates a reduction in the energetic cost of running, allowing runners to achieve higher speeds at the end of the maximal incremental treadmill test without significant increases in the VO_2_max. There is also some evidence that improvements in high-intensity aerobic performance are not strongly associated with improvements in VO_2_max since low responders for one parameter are not necessarily low responders for another (Vollaard et al., 2009). Moreover, greater efficiency at moderate and high loads in a maximal progressive short effort could indicate the effectiveness of training, but its translation to a specific performance (e.g., half-marathon) is of less importance, since the speeds of running a half-marathon are lower than those at which the improvement has occurred.

Our results also indicated that focusing on other specific tests in the field could be a better tool in assessing the improvement in endurance elicited by training programs. Thus, in both groups, maximal incremental tests in the field showed that after the 12-week training period, most of the participants increased their TTE, exhibiting a homogeneous response (Figure 1C) and significantly higher mean times (Table 1). However, the two groups showed different behaviors in their responses for the interval series (power) and long run (pace) field tests (Figure 2). For both tests, there was a greater number of responders among the women in the HIIT group than in the MICT group. This could be due to the fact that the HIIT sessions of short-burst intervals interspersed with low-intensity recovery periods lead to a strong engagement of neuromuscular and musculoskeletal systems (improving power in 200-m and 400-m series), allowing individuals to run at high intensities with low lactate levels (increasing pace during long runs; see for review, Buchheit and Laursen, 2013). The heterogeneity in the responses and the different behaviors in the MICT and HIIT groups reported here for middle-aged women are consistent with those recently found for young males and females (Bonafiglia et al., 2016) and also with the findings of a multi-center comparison study of trainability by Williams et al. (2019) involving a big sample size with different ages and conditions.

The different individual responses to the training interventions, with some subjects as *responders* and others as *non-responders*, may be a consequence of a combination of two factors. First, all the women participating in this study were amateur runners with some level of previous training experience. This previous high or near maximal physical fitness could have affected the changes in the performance indicators analyzed in some participants, since diminished improvements in performance indicators such as VO_2_max have been described in trained subjects enrolled in training programs, in contrast to the rapid increases observed in untrained individuals (Wenger and Bell, 1986). Second, responses to training might be highly individual, as has been recently described for HIIT by Astorino et al. (2018), who found that some participants showed meaningful increases in some performance variables, whereas others showed no changes in their previous values. Several anatomical, biochemical and physiological systems interact to influence sports performance and could account for this inter-individual variability to training. The individual differences in metabolic pathways could quantitatively increase or decrease the measured parameters and induce synergistic or antagonistic effects depending on the training protocol. For example, it is well known the human individual variation in skeletal muscle fiber-type proportion (Simoneau and Bouchard, 1989) which is highly correlated with sports performance (Costill et al., 1976). Other factor involved in the variability of the responses to training could be the metabolic and biomechanical specificity of each training program (Kenney et al., 2012). The different constraints imposed by MICT or HIIT would result in more or less evident changes in performance depending on the characteristics of each training schedule and the parameters assessed. This could explain finding more responders in the HIIT group in the interval run series (Figure 2A).

Our results provide further support for individualized exercise prescription to optimize workouts. Furthermore, our findings suggest that several performance indicators can be used to assess training programs. For instance, the analysis of continuous variables throughout a training process beyond maximal values could provide more sensitive information to determine specific adaptations in different training programs. This has been recently demonstrated by Garcia-Retortillo et al. (2019), who compared HIIT and MICT programs and observed that, despite improving markers of aerobic fitness to a similar extent, changes in cardiorespiratory coordination were specific for each training intervention. For endurance recreational runners, moderate continuous training with extensive aerobic loads is the most frequently used type of training (see for example, Fokkema et al., 2020). However, a huge amount of time is invested in training and the protocols are repetitive, increasing the risk of musculoskeletal injuries due to overuse (Dias-Lopes et al., 2012). In fact, one of the initial participants following MICT abandoned the study suffering from an injury. Conversely, high intensity training with low repetition not only reduces training time, but also decreases the risk of injuries due to overuse, as has been recently reported by Mallol et al. (2020). In the present work, we did not report any injuries in HIIT ruling out the possible increased risk of acute injury due to the greater intensity of the HIIT program.

The main strength of this study was the homogeneity of the group studied, i.e., middle aged women with previous amateur experience in running events. This population group is poorly studied and has become increasingly involved in amateur running events in recent years (van Dyck et al., 2017). The main limitation of the study was the small sample size of *n* = 20, with *n* = 10 in each training group. It would have been better to have a greater sample size, which, undoubtedly, would have rendered higher power to the conclusions and provided more sensitivity to detect significant differences in the eventual changes. Similar studies could be performed in the future in population groups with different genders and/or ages, as well as using participants with different levels of fitness (from sedentary to elite athletes). Our idea for future studies is to design experimental work that can identify in advance responders and non-responders with the aim of creating a pre-training schedule that can be modified to help non-responders improve their performance. In this sense, incorporating complex system approaches, such as those reported by Balagué et al. (2016) and Garcia-Retortillo et al. (2017) on cardiorespiratory coordination, will be of great value in assessing a strategic research framework for individual training prescriptions.

## Conclusion

Two different training programs for a half-marathon, one based on high intensity and moderate training volume (HIIT) and the other involving moderate intensity and greater training distances and times (MICT), were compared in a group of amateur middle-aged women runners. A global improvement in the mean finishing time for the half-marathon and improvements in field performance indicators at the end of the training period, such as TTE, power in short-run series and long run pace, were observed in the two training groups. However, there were different inter-individual responses after the training period, with some participants showing improved performance (*responders*) and others failing to exhibit positive changes (*non-responders*). These different responses depended on the training group, with more heterogeneous results in HIIT group. As a future perspective, these findings could help running coaches and amateur running practitioners to modify workloads to optimize performance. Compiling data on individual measurements (such as anthropometrical, epidemiological, physiological, and those regarding to performance), a predictive model could be constructed with the goal of deciding the suitability of the training protocol to be applied. Depending on the runner’s vital status, a MICT, HIIT or a mixed training model could be prescribed, optimizing runner’s effort and time dedicated to training, which would decrease injury risk factors and improve training adherence.

## Data Availability Statement

The raw data supporting the conclusions of this article will be made available by the authors, without undue reservation.

## Ethics Statement

The studies involving human participants were reviewed and approved by Institutional Ethics Committee of the University of Barcelona (Institutional Review Board number, IRB00003099). The patients/participants provided their written informed consent to participate in this study.

## Author Contributions

CJ and JT conceived and designed the study. JB and CJ conducted the experiments. JB, CJ, and JT analyzed the data and wrote the draft manuscript. JV, JM, GV, and TP corrected the draft manuscript and contributed the analytical tools. All authors read and approved the manuscript.

## Conflict of Interest

The authors declare that the research was conducted in the absence of any commercial or financial relationships that could be construed as a potential conflict of interest.
